# Elastic metamaterials for independent realization of negativity in density and stiffness

**DOI:** 10.1038/srep23630

**Published:** 2016-03-23

**Authors:** Joo Hwan Oh, Young Eui Kwon, Hyung Jin Lee, Yoon Young Kim

**Affiliations:** 1 Institute of Advanced Machine and Design, Seoul National University, 599 Gwanak-ro, Gwanak-gu, Seoul, 151-744, Korea; 2 Korea Institute of Nuclear Safety, 62 Gwahak-ro, Yuseoung-gu, Daejeon 305-338, Korea; 3 Department of Mechanical and Aerospace Engineering, 599 Gwanak-ro, Gwanak-gu, Seoul, 151-744, Korea

## Abstract

In this paper, we present the first realization of an elastic metamaterial allowing independent tuning of negative density and stiffness for elastic waves propagating along a designated direction. In electromagnetic (or acoustic) metamaterials, it is now possible to tune permittivity (bulk modulus) and permeability (density) independently. Apparently, the tuning methods seem to be directly applicable for elastic case, but no realization has yet been made due to the unique tensorial physics of elasticity that makes wave motions coupled in a peculiar way. To realize independent tunability, we developed a single-phased elastic metamaterial supported by theoretical analysis and numerical/experimental validations.

Interest in negative material properties has initially grown in electromagnetic field^1^,^2^,^3^, being realized by metamaterials. Since then, several interesting applications in antennas, lenses, wave absorbers and others have been discussed. The advances in electromagnetic metamaterials are largely due to independent tuning of the negativity in permittivity and permeability. As done in electromagnetic waves, negative material properties can be also independently tuned in acoustics^4^,^5^,^6^,^7^,^8^ by the analogy between electromagnetic and acoustic waves. Here, our interest is on elastic waves in solid media. We aim to realize solid elastic metamaterials the material properties of which can be independently tuned to be negative for uni-axial wave propagation. In spite of great potentials of single- and double-negative solid metamaterials useful for super vibration shielding^9^,^10^,^11^,^12^, over-the-diffraction-limit ultrasonic imaging^13^,^14^,^15^,^16^ and elastic cloaking^17^,^18^,^19^, there is no realization to independently tune density and stiffness in solid elastic media.

In that both acoustic and elastic waves propagate by particle motions or oscillations, one may immediately conjecture that the independent tuning method of negativity in material property developed for acoustic waves can be directly used in problems dealing with elastic waves. However, due to the unique tensorial physics of elasticity, complex coupling occurs among various deformation modes such as longitudinal, bending and shear motions. Therefore, the ideas from electromagnetic/acoustic waves cannot be directly used. Earlier investigations^20^,^21^,^22^,^23^,^24^,^25^,^26^,^27^ in elastic metamaterials showed that a monopole or dipole resonance mode of a co-axial internal resonator can be utilized to make bulk modulus or density negative. Among them, Liu *et al*.^25^ and Bigoni *et al*.^26^ proposed chiral elastic metamaterials consisting of solid materials and Zhu *et al*.^27^ realized a single-phased elastic metamaterial. Dubois *et al*.^28^,^29^ realized flat lens and superoscillations with double-negative elastic metamaterials. Hou *et al*.^30^ studied elastic metamaterial having tunable negative stiffness. The limitation of these investigations is, however, the lack of independent tunability of negative stiffness and density because any change in the resonator configuration simultaneously varies the resonance frequencies governing negative stiffness and density. Recently, Lai *et al*.^31^ presented the simulation results for metamaterial that independently controls negative stiffness and density by multi-phased resonators. However, the realization of an elastic metamaterial having independent tunability of negative stiffness and density with experimental verification has not been achieved.

Here, we present the first realization of an elastic metamaterial allowing independent negativity tuning in stiffness and density. To this end, a single-phased elastic metamaterial in Fig. 1 is developed. As in earlier investigations^20^,^21^,^22^,^23^,^24^,^25^,^26^,^27^, we use resonance modes for the tuning. However, the uniqueness in the present study is that the unit cell of the proposed metamaterial involves two single-phased independent resonators each of which realizes negative density or stiffness. The elastic wave in consideration is the lowest symmetric Lamb wave mode uni-axially propagating along the *x* direction, i.e., the S0 wave mode the dominant motion of which is in the propagating direction^32^. Referring to Fig. 1, the metamaterial has so-called *x*- and *y*-resonating parts which have local resonance modes in the *x*-direction (propagation direction) and in the *y*-direction (direction perpendicular to the propagation direction), respectively. While more details of the involved physics will be given later, the negative density and stiffness are realized by the *x*- and *y*-resonating parts, respectively. Since the resonance frequencies of the two resonators can be independently tuned, independent tuning of negativity in stiffness and density is available and single or double negativity for a selected range of frequencies can also be achieved. Especially, in realizing negative stiffness by the *y*-resonating parts, the coupling of different deformation modes, longitudinal and bending, is elaborately used; the counterpart of the coupling cannot be found in electromagnetic or acoustic problems. Specifically, the slender members can exhibit both longitudinal and shear-bending motions, unique coupling phenomena in elasticity governed by the tensor field. Later, we will show how the coupling affects negative stiffness behavior. In the present realization of the metamaterial, the inclinations of slender members connected to the central rectangular mass in the *y*-resonating part make the coupling effect which plays a key role for the stiffness tuning.

## Results

**Figure 1:**
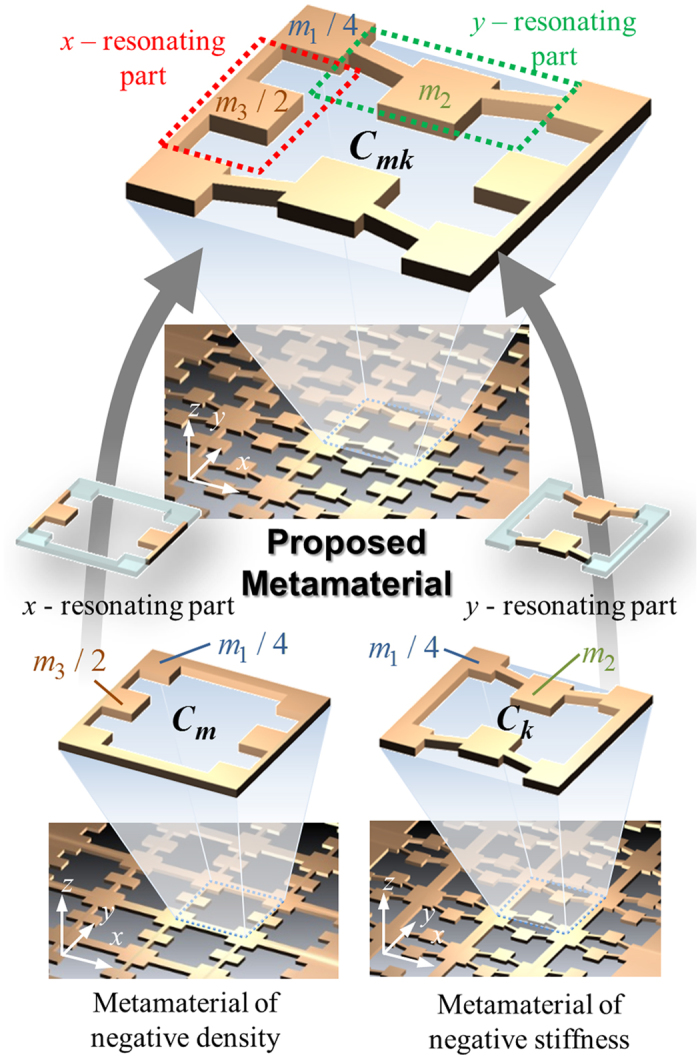
The sketch of the proposed unit cell *C*_*mk*_ which can be constructed by two independent unit cells *C*_*m*_ and *C*_*k*_ realizing negative effective mass and stiffness along the *x* direction, respectively.

**Figure 2:**
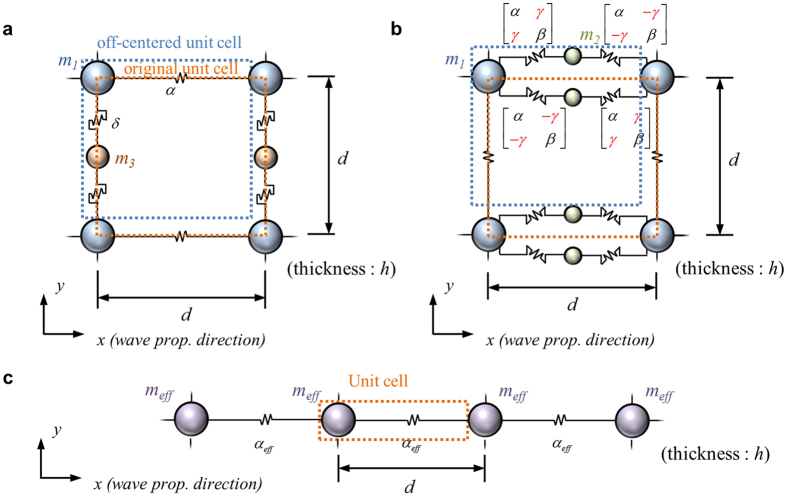
The mass-spring unit cell model corresponding to (**a**) the unit cell ***C***_***m***_ for negative density, (**b**) the unit cell ***C***_***k***_ for negative stiffness and (**c**) a periodic mass-spring system under uni-axial wave motion in the *x*-direction.

To illustrate our idea clearly, we will consider the metamaterial in Fig. 1. As Fig. 1 suggests, the square unit cell (***C***_***mk***_) of the metamaterial has two resonating parts the physics of which can be better investigated by square unit cells ***C***_***m***_ and ***C***_***k***_ in Fig. 1. Here, the symbols ***C***_***m***_ and ***C***_***k***_ denote the unit cell exhibiting negative mass and stiffness, respectively, for the S0 wave mode propagating in the *x* direction. Because the mechanics of the unit cell ***C***_***mk***_ can be viewed as a combination of ***C***_***m***_ and ***C***_***k***_ as illustrated in Fig. 1, how the negativities are realized by ***C***_***m***_ and ***C***_***k***_ will be investigated first. To model the mechanics of ***C***_***m***_ and ***C***_***k***_ in association with the S0 wave propagating in the *x* direction, the elastic metamaterial solids forming ***C***_***m***_ and ***C***_***k***_ will be replaced by the mass-spring system shown in Fig. 2(a,b). Since we are mainly focused on the S0 wave mode, only particle motion on the *x-y* plane will be considered in the mass-spring system. Although the S0 wave mode is a 3-dimensional wave phenomenon, its characteristics can be analyzed with a two-dimensional model at sufficiently low frequencies^14^,^15^,^27^.

### Identification of effective mass and stiffness

To begin with, we review the well-known dispersion equation and expression of characteristic impedance in a simple one-dimensional periodic mass-spring system in Fig. 2(c). The system consists of an infinite number of periodically-arranged lumped masses of mass 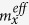 and springs of stiffness 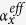. Starting from the forces acting on 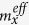, the dispersion equation can be derived as^33^ (see the Supplementary Material for more detailed derivation)


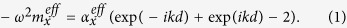


Where *ω, k* and *d* denote the angular frequency, the wavevector and the period, respectively.

To uniquely define the effective mass and stiffness from the dispersion relation, the expression for characteristic impedance is also needed. The characteristic impedance Z of the periodic mass-spring system shown in Fig. 2(c) is written as^33^ (see the Supplementary Material for more detailed derivation)


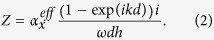


Where *h* is the thickness of the unit cell.

As can be seen in equation (2), one can determine the effective stiffness of a metamaterial system by writing its characteristic impedance equation and comparing the result with equation (2). Then, the effective mass can be determined by comparing the dispersion equation of a system of interest with equation (1).

### Analysis of effective mass tunable sub unit cell

To introduce a method to tune effective mass alone, let us consider the mass-spring model shown in Fig. 2(a) representing ***C***_***m***_. As explained, although the S0 wave mode mainly considered in this work is a 3-dimensional wave phenomenon, it can be accurately analyzed with a two- dimensional mass-spring system if the operating frequency range is sufficiently low as in this work^14^,^15^,^27^. Here, *α* denotes the longitudinal spring coefficient of the beam segment connecting masses *m*_1_’s and *δ* denotes the shear-bending spring coefficient of the beam segment connecting mass *m*_1_ and *m*_3_. To facilitate the analysis, the square unit cell is slightly off-centered but the resulting dispersion curves will not be affected because of periodicity of the unit cells. Following the detailed procedures in the Supplementary Material, the wave dispersion equation and the impedance equation of the mass-spring system in Fig. 2(a) can be obtained as


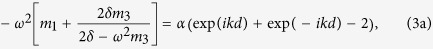



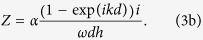


Comparing equations (3a,b) with equations (1, 2), the effective mass and stiffness can be identified as


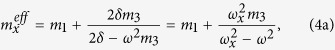



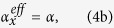


where 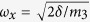 corresponds to the resonance frequency of the *x*-resonating part when *m*_3_ oscillates in the *x* direction. If *ω* is much lower than *ω*_*x*_, the effective mass simply becomes the total mass, *m*_1_ + *m*_3_. However, for *ω* near *ω*_*x*_, the motion of the *x*-resonating part significantly affects the effective mass, making it negative when *ω* becomes slightly larger than *ω*_*x*_. The equation (4a) to calculate the effective mass looks similar to the expression obtained earlier with an elastic metamaterial using a coaxial internal resonator^34^ if *ω*_*x*_ is replaced by its dipole resonance frequency. It is worth remarking that in the present case, the motion of the resonating part involves bending deformation of slender members mainly and interacts with the main motion of *m*_1_, thus avoiding a need to use multi-phased materials as used to make the coaxial resonators.

### Analysis of effective stiffness tunable sub unit cell

Let us now derive the effective mass and stiffness for the metamaterial made of ***C***_***k***_ by using the discrete model in Fig. 2(b). Note that ***C***_***k***_ should be so designed that we can tune, independently from *ω*_*x*_, the resonance frequency to be associated with negative stiffness. Here, our proposition is to utilize coupling between *x* and *y* directional motions by intentionally inclining the slender members connecting *m*_1_ and *m*_2_. The resulting coupling phenomenon between the longitudinal and shear-bending motions is unique in the tensorial physics of elasticity. The coupled stiffness is denoted by *γ* in Fig. 2(b) which represents the coupling effect between *x* and *y* directional motions. Thus, the spring coefficients of the slender members can be expressed in the following relations:


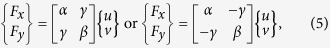


where *u* and *v* denotes the *x*- and *y*-directional displacements applied to the coupled spring. Referring to the original continuum configuration of ***C***_***k***_ shown in Fig. 1, one can see why the −*γ* term also appears; the two symmetric members with the opposite inclination angles are used to connect *m*_1_ and *m*_2_. As a result, not only the *x-*directional but also the *y*-directional motions of *m*_2_ should be considered in deriving equations of motion.

Following the detailed procedures in the Supplementary Material, the wave dispersion equation and the impedance equation of the mass-spring system in Fig. 2(b) can be obtained as


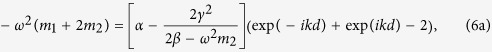



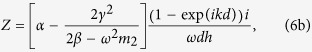


for which the following assumption is used,


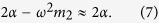


The assumption in equation (7) can be valid because the operating frequency of interest is assumed to be much lower than 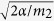. (Also note that *α* is typically one order larger than *β* as demonstrated in Table S1 in the Supplementary Material). The frequency 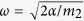 corresponds to the Bragg gap frequency^33^ in which the wavelength is almost half the unit cell size. Thus, the assumption in equation (7) holds when the wavelength *λ* is much larger than the unit cell size *d* (say, when *λ* > 4*d*). Because the unit cell size is very small compared with the wavelength in the matrix (*λ*_*matrix*_ > 6*d*) in the frequency range below 40 kHz, the use of the assumption can be justified as can be seen in Figs S6 and S7 in the Supplementary Material.

Finally, the effective mass and stiffness are identified as, by comparing equations (6a,b) with equations (1, 2),


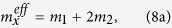



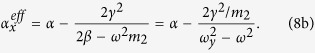


Where 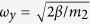 is the resonance frequency of the *y*-resonating part as it oscillates in the *y* direction. Equation (8) shows that the *y*-resonating part affects the effective spring coefficient only while making the effective mass unaffected by *ω*_*y*_. Obviously, the effective stiffness 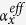 becomes negative at frequencies slightly lower than *ω*_*y*_. It is remarked that there were some earlier attempts, without actual realization or experiment, to utilize *y*-directional motions^21^ or a nonlinear phenomenon^35^ to control the *x*-directional stiffness.

### Metamaterial consisting of independently-tuned mass and stiffness

From the previous analytic investigations, the effective mass and stiffness for the metamaterials having only the *x*- and *y*- resonating part are identified. Because the metamaterial consisting of the unit cell ***C***_***mk***_ in Fig. 1 can be viewed as the combination of ***C***_***m***_ and ***C***_***k***_, the wave dispersion equation and the impedance equation can be written as


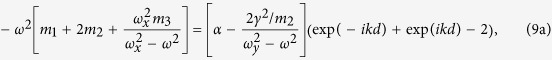



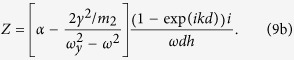


Accordingly, the effective mass and stiffness for the metamaterial can be written as


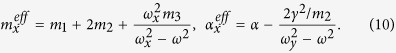


Equation (10) reveals that our goal to realize independently-tunable effective mass and stiffness can be indeed achieved by the newly-proposed elastic metamaterial. Because *ω*_*x*_ and *ω*_*y*_ are independently tunable, one can realize single- or double-negative elastic metamaterials for different ranges of frequencies.

The effective mass and stiffness derived above can be validated directly or indirectly with numerical methods. As an indirect way, one can compute the dispersion curve by using the one-dimensional dispersion equation (1) with the effective mass and stiffness in equations (4 and compare it with the dispersion curve obtained for the original solid unit cell shown in Fig. 1. A direct approach is to estimate the effective parameters by the retrieval method^36^ developed for elastic metamaterials^37^. The direct and indirect numerical validations are given in the Supplementary Material which also include the actual geometric data of the unit cells ***C***_***m***_, ***C***_***k***_ and ***C***_***mk***_.

### Deformation mechanism of negative parameters

**Figure 3:**
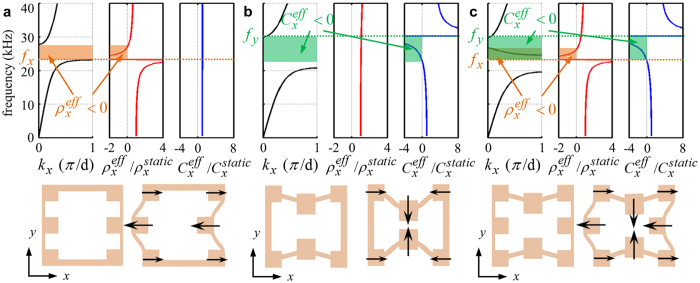
The *x*-directional dispersion curve, effective parameters and mode shapes of the proposed unit cells (**a**) ***C***_***m***_ (**b**) ***C***_***k***_ and (**c**) ***C***_***mk***_.

Let us now investigate, in some details, the dispersion curves and frequency dependences of *x*-directional effective density 
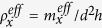
 and modulus of elasticity 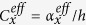 where *h* is the unit cell’s thickness and *d*, its width and height. As clearly shown in Fig. 3(a,b), the metamaterials made of ***C***_***m***_ and ***C***_***k***_ exhibit single negativities in density and stiffness near *f*_*x*_ (=*ω*_*x*_/2*π*) and *f*_*y*_ (=*ω*_*y*_/2*π*), respectively, which are also confirmed by the dispersion curves^38^ showing the formation of stop bands.

The sketches of the deformation patterns of ***C***_***m***_ and ***C***_***k***_ around or near *f*_*x*_ and *f*_*y*_, respectively, show how the negativity in density and stiffness is realized. In Fig. 3(a), *m*_1_, the main mass parts through which the unit cells are connected to each other, moves to the right while *m*_3_ moves to the left. Thus, the total momentum of ***C***_***m***_ becomes negative because of the 180° out-of-phase motion of *m*_3_ for a positive velocity of the unit cell, causing the effective density negative. On the other hand, Fig. 3(b) shows the mode shape of the unit cell ***C***_***k***_ sketched at a frequency just below the resonance frequency *f*_*y*_. Because of the large up- and down-ward *y*-directional motions of the *m*_2_ parts under a force (motion) at the left side of ***C***_***k***_, the right side of ***C***_***k***_ moves to the left, the opposite direction to the force (motion) at the left side. Thereby, the effective stiffness becomes negative. This is possible because of the elaborate coupling of *x* and *y* directional deformations which cannot be found in electromagnetic or acoustic wave cases.

The metamaterials consisting of ***C***_***mk***_, which is the combination of ***C***_***m***_ and ***C***_***k***_, exhibit the combined effects of the two independent metamaterials made of ***C***_***m***_ and ***C***_***k***_, as clearly demonstrated in Fig. 3(c). This finding indeed confirms the independent tunability of the proposed metamaterial in its density and stiffness values. As being obvious, there appears a passing zone with the negative group velocity (i.e. the negative slope in the dispersion curve) in the zone of overlapping frequencies of the negative density in ***C***_***m***_ and the negative stiffness in ***C***_***k***_. Because *f*_*x*_ and *f*_*y*_ can be independently tuned in the developed metamaterial, the metamaterials can be tailored to meet specific applications requiring single and/or double negativity.

### Refractive index and Impedance of the metamaterial

In this section, the analytic approach on the refractive index and the impedance of the metamaterial will be carried out. One can calculate these parameters directly from equations (9a,b) but the periodicity condition used for these equations would make it difficult to compare the parameters with those of natural materials. Accordingly, we mainly consider evaluating these parameters for low wavevectors, i.e., *kd* ≪ 1. In this range, one can assume that 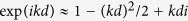 and equations (9a,b) can be re-written as


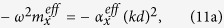



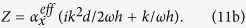


Considering that 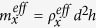 and 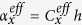, one can derive^33^ the following expressions for the phase velocity *v*_*p*_ and the impedance Z:


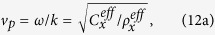



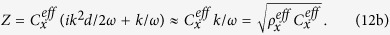


In the proposed metamaterial, the effective density and stiffness, 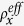 and 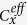, can be tuned from negative infinity to positive infinity as in Fig. 3(c). Thus, one can achieve any refractive indices (including negative values) and impedances by using the proposed metamaterial. Also, it is easy to realize the desired refractive index and impedance values by using the proposed metamaterial because the effective density 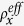 and stiffness 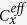 can be independently tuned. This analysis suggests that the proposed metamaterial can be widely applied in various wave devices. The imaginary phase velocity obtained from single negativity can be effectively used in vibration shielding^9^,^10^,^11^,^12^. Also, the negative or imaginary phase velocity can be applied to realize elastic superlens or hyperlens for sub-wavelength resolution^13^,^14^,^15^,^16^. Moreover, the impedance tunability of the metamaterial can be applied in various impedance matching or wave filtering applications to control the wave energy transmission.

### Realization and experiments

**Figure 4:**
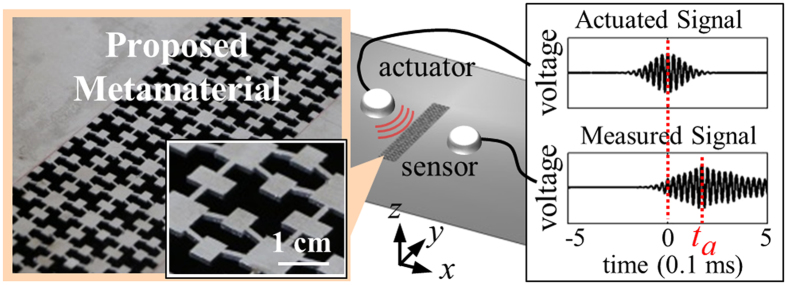
Experimental setting for wave propagation through the developed metamaterials with a photo of the fabricated metamaterial. The detailed geometries of the unit cells are given in Fig. S1.

Finally, the metamaterials are fabricated and their wave characteristics are experimentally investigated. Figure 4 illustrates the experimental setup and also shows a sample of transmitted and measured signals for the experiments. Three sets of experiments were performed with the metamaterials consisting of ***C***_***k***_, ***C***_***m***_ and ***C***_***mk***_, which are made of an aluminum only. Since these metamaterials are made of a single-phased low-loss aluminum, adverse effects of loss can be insignificant.

**Figure 5:**
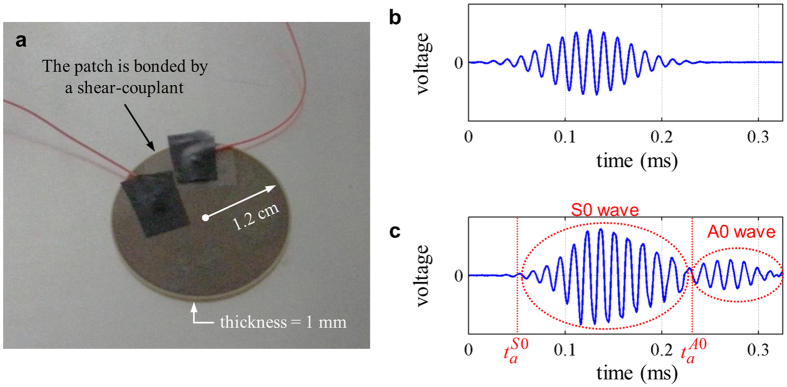
(**a**) The piezoelectric transducer installed on an aluminum plate. (Note that a heavy mass is placed on it during experiments). (**b**) Actuated input signal and (**c**) measured elastic wave signal from the transducer installed on a 1 mm thick homogeneous aluminum plate (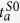: analytically calculated arrival time of S0 wave mode, 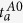: analytically calculated arrival time of A0 wave mode in aluminum plate).

In the experiments, the S0 wave was actuated by the actuating piezoelectric transducers (thickness: 1 mm, radius: 1.2 cm, illustrated in Fig. 5(a)). We used the modulated Gaussian pulses centered at 15, 25 and 35 kHz as input signals. To make sure that the dominant wave mode generated and measured with the used patch-type piezoelectric transducers is the S0 wave mode, not the undesirable A0 mode (the lowest anti-symmetric Lamb wave) in the frequency range of interest, a reference pitch-catch experiment was performed in a homogeneous aluminum plate. The input signal to actuate the patch-type piezoelectric transducer is shown in Fig. 5(b) and the measured output signal by a receiving patch-type transducer that is 30 cm apart from the actuating transducer is plotted in Fig. 5(c). Because the transducer is so configured as to predominantly generate the S0 wave mode^39^ and the group velocity of the S0 wave mode in the aluminum plate (
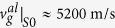
) is much faster than the group velocity of the A0 wave mode in the aluminum plate (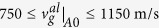 in the frequency range of interest), we can use the first arrival signal of the S0 wave mode which can be completely distinguishable from the A0 wave mode. Inside the metamaterial, the difference in the group velocity between the S0 and A0 wave modes is more severe, which makes it easier to work only with the S0 wave mode. It was found that 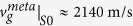 while 
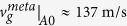
. Also, since the A0 wave mode of the metamaterial made of ***C***_***mk***_ has a stop band at around 25 kHz, only the S0 wave mode is measured at the double negative region of the metamaterial made of ***C***_***mk***_. Because we can capture the first arrival pulse of the S0 wave mode only in actual experiments, the resulting data analysis is performed with the S0 mode.

**Figure 6:**
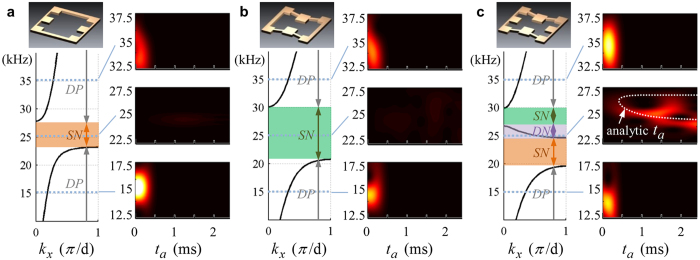
Comparison between experimental and analytic results (*k*_*x*_: wave number in the *x* direction, *t*_*a*_: arrival time) for the metamaterials consisting of (**a**) ***C***_***m***_ (**b**) ***C***_***k***_ and (**c**) ***C***_***mk***_. Here, DP, SN and DN stand for double positivity, single negativity and double negativity, respectively. In (**c**), the white dotted-line denotes the analytically calculated arrival time.

After S0 wave is generated from the actuating piezoelectric transducer, the transmitted S0 wave through the metamaterials consisting of ***C***_***k***_, ***C***_***m***_ and ***C***_***mk***_, respectively, is measured from the sensing piezoelectric transducer. Since the metamaterials are dispersive^40^, the measured signals were analyzed through the Short-Time Fourier Transform (STFT). Figure 6 shows the experimental results; the detailed experimental procedures, the process of STFT and meaning of the color level in Fig. 6 can be found in the Supplementary Material.

Figure 6 shows that the elastic metamaterial having only the *x*- or *y*-resonating part exhibits no wave transmission in the frequency ranges in which either the effective density or the effective stiffness is negative; see Fig. 6(a,b). Using the well-known fact that waves cannot propagate through a metamaterial with either the negative effective density or stiffness, one can immediately see from Fig. 6(a,b) that the metamaterials made only of ***C***_***k***_ or ***C***_***m***_ has single negativity around 25 kHz. At the excitation frequency of 25 kHz lying between *f*_*x*_ and *f*_*y*_, the wave is transmitted through the metamaterial made of ***C***_***mk***_ as confirmed in Fig. 6(c), showing that the metamaterial made of ***C***_***mk***_ has double positivity or double negativity.

To further investigate the transmitted waves, analytically calculated arrival times *t*_*a*_ are plotted by the white dotted line in Fig. 6(c). As can be shown in Fig. 6(c), the arrival times predicted by the analytic approach show good agreement with the experimental results. This means that the experimentally measured group velocities are almost identical to the analytically calculated group velocities. At around 27 kHz, however, little transmission occurs because of a large impedance mismatch between the metamaterial and the aluminum plate. Also, the second arrival pulse component of 25 kHz appears around 1.5 ms due to internal reflections within the metamaterials and its appearance cannot be predicted by the analytic analysis. These results show that the independent achievement of negative stiffness and density is realized by the proposed metamaterials. More experimental results showing the independent tunability can be found in the Supplementary Material.

### Experimental measurement of the negative phase velocities

**Figure 7:**
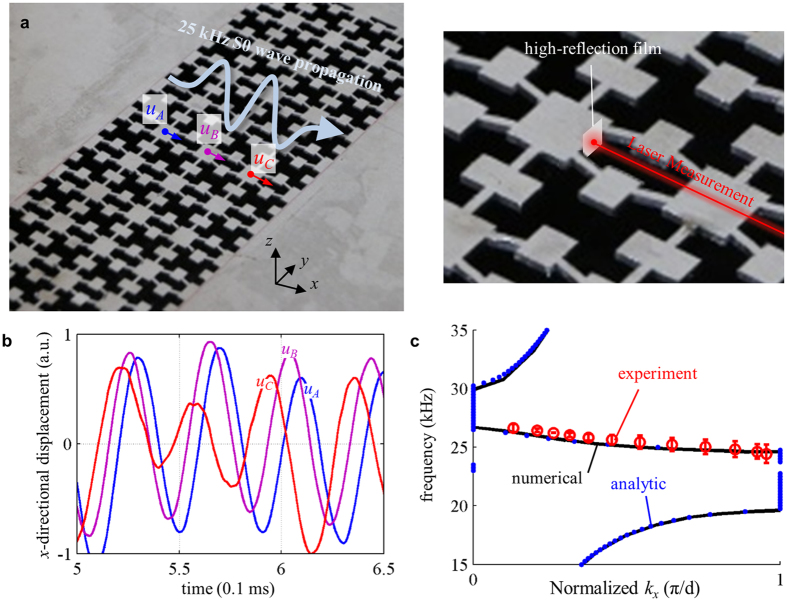
(**a**) Experimental setting to measure the phase velocity inside the metamaterial made of ***C***_***mk***_, (**b**) experimentally measured *x*-displacements (*u*_*A*_, *u*_*B*_ and *u*_*C*_) by a laser vibrometer, and (**c**) dispersion curves by analytic, numerical and experimental predictions. (The curves are plotted for positive wavevectors).

The experimental results shown in Fig. 6 support the formation of single negativity and now we will experimentally show the formation of double negativity of the metamaterial made of ***C***_***mk***_, i.e., the negative phase velocity at 25 kHz. Figure 7(a) shows the schematic figure of the experimental setup to measure the displacements *u*_*A*_, *u*_*B*_ and *u*_*C*_ at three different points, *A*,*B* and *C*, inside the metamaterial. For the experiment, a thin highly-reflective rectangular film was vertically installed at the measurement locations. Then a laser vibrometer (OFV-551, Polytec) was used to measure the *x*-directional displacement. For the actuation, the same piezoelectric transducer shown in Fig. 5(a) is used.

Figure 7(b) shows the measured *x*-directional displacement *u*_*A*_, *u*_*B*_ and *u*_*C*_. From Fig. 7(b), it can be clearly seen that the wave peaks move backwards, indicating negative phase velocity of the metamaterial made of ***C***_***mk***_ around 25kHz. Also, the experimentally measured displacement fields match well with those obtained with numerical simulation in the Supplementary Material (shown in Fig. S5(c)). The magnitude difference between the displacements from the simulation and the experiment is due to the difficulty to install the thin film exactly vertically. Nevertheless, the experimental measurements clearly reveal that the phase velocity in the metamaterial at 25 kHz that belongs to the double negative zone is negative.

We also evaluated the wave dispersion curve of the metamaterial from the experimental measurement. To plot the dispersion curve from the experimental data, the measured displacements were post-processed by the Fourier transform to obtain the phase difference between each measurement point. To minimize statistical errors, measurements were made at various points. Then, the wave vector was calculated for various frequencies around 25 kHz. Figure 7(c) compares the experimentally evaluated and the numerically calculated dispersion curves. The statistic errors indicated by the error bar in Fig. 7(c) are mainly due to the fabrication imperfection in the metamaterial geometry. Note that the experimentally measured wavevectors are all negative values although they are plotted in the positive wavevector domain for the comparison in Fig. 7(c). Very good agreements between two results validate our theoretic investigations performed earlier.

## Conclusion

This study presents the first metamaterial realization with independent tuning of effective negative density and stiffness with a single-phased material. In spite of apparent similarity between elastic waves and electromagnetic/acoustic waves, the independent negativity tuning in elastic metamaterials has not been realized earlier. To realize the independent negativity tuning, a single-phased elastic metamaterial was proposed. Here, among others, the independent-tunable negative stiffness was realized by a locally-resonating part the motion of which is dominant in the direction perpendicular to the wave propagation direction. The theoretical and experimental wave analyses of the metamaterials were carried out to confirm the independent tunability of negativity, with realizations of single negative density/stiffness and simultaneous negativity in density and stiffness. Considering many practical important applications of elastic waves in ultrasonic imaging, vibration shielding, etc., our metamaterials could lead to active explorations in elastic metamaterials, which currently appear to be less active in the community. However, the extensions of the proposed 1-dimensional metamaterial for the realization of 2- or 3-dimensional metamaterials would require further studies because of more complicated coupling of longitudinal and shear waves in higher dimensions.

## Additional Information

**How to cite this article**: Oh, J. H. *et al*. Elastic metamaterials for independent realization of negativity in density and stiffness. *Sci. Rep.* **6**, 23630; doi: 10.1038/srep23630 (2016).

## Supplementary Material

Supplementary Information
