# Supplementary material for: Elastic metamaterials for independent realization of negativity in density and stiffness
Source: Sci Rep. 2016 Mar 23;6:23630. doi: 10.1038/srep23630 (PMC4804290; doi:10.1038/srep23630)
Supplement: Supplementary Information [file srep23630-s1.pdf]

Supplementary Materials

Elastic metamaterials for independent realization of negativity in density and stiffness

Joo Hwan Oh[a], Young Eui Kwon[b], Hyung Jin Lee[c] and Yoon Young Kim[a, c*]

[a]*Department of Mechanical and Aerospace Engineering, Seoul National University, 599 Gwanak-ro, Gwanak-gu, Seoul, 151-744, Korea*

[b]*Korea Institute of Nuclear Safety, 62 Gwahak-ro, Yuseoung-gu, Daejeon 305-338, Korea*

[c]*Institute of Advanced Machine and Design, Seoul National University, 599 Gwanak-ro, Gwanak-gu, Seoul, 151-744, Korea*

Analytic investigation of the simple periodic mass-spring system

First, the simple periodic mass-spring system consisting of m_x^{eff} and springs of stiffness α_x^{eff}, as shown in Fig. S1, is considered. Starting from the forces acting on m_x^{eff}, the equation of motion can be derived as

$$m_x^{eff}\frac{\partial^2 u_n}{\partial t^2}=\alpha_x^{eff}(u_{n+1}-u_n)-\alpha_x^{eff}(u_n-u_{n-1}). \quad \text{(S1)}$$

where u_n is the x-directional displacement of the nth mass and t denotes time. Assuming time harmonic wave motion at an angular frequency of ω, u_n can be expressed as

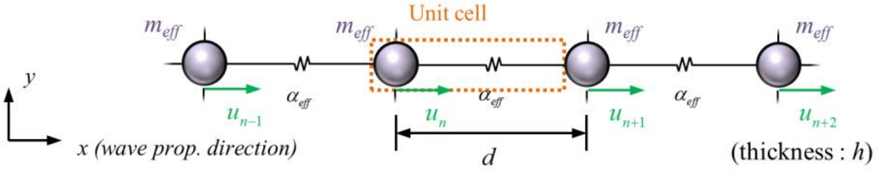

Fig. S1. A periodic mass-spring system under uni-axial wave motion in the x-direction.

* Corresponding Author, Professor, yykim@snu.ac.kr, phone +82-2-880-7154, fax +82-2-872-1513

$u_n = U_n \exp[i(\omega t - kx)]$ ($i = \sqrt{-1}$) where k denotes the wavenumber. Noting that $u_{n+1} = \exp(-ikd)u_n$ and $u_{n-1} = \exp(ikd)u_n$ for a periodic system, equation (S1) yields the following dispersion equation,

$$-\omega^2 m_x^{eff} = \alpha_x^{eff}(\exp(-ikd) + \exp(ikd) - 2) . \tag{S2}$$

To uniquely define the effective mass and stiffness from the dispersion relation, the expression for characteristic impedance is also needed. Using the force F exerted on the $(n+1)^{\text{th}}$ mass by the nth mass,

$$F = \alpha_x^{eff}(u_n - u_{n+1}) = \alpha_x^{eff}(\exp(ikd) - 1)u_{n+1} , \tag{S3}$$

the characteristic impedance Z is written as

$$Z = \frac{\sigma_{xx}}{\partial u_{n+1} / \partial t} = \frac{F / dh}{\partial u_{n+1} / \partial t} = \frac{F}{i\omega dh u_{n+1}} = -\frac{Fi}{\omega dh u_{n+1}} = \alpha_x^{eff} \frac{(1 - \exp(ikd))i}{\omega dh} . \tag{S4}$$

where σ_{xx} is the normal stress in the x direction.

Analytic investigation of the metamaterial with the unit cell $\boldsymbol{C_m}$

The mass-spring system of the metamaterial with the unit cell $\boldsymbol{C_m}$ is shown in Fig. S2. To facilitate the analysis, the square unit cell is slightly off-centered but the resulting dispersion curves will not be affected because of periodicity of the unit cells. Referring to the off-centered unit cell in Fig. S2, the x-directional displacements of m_1 and m_3 in the $(n, j)^{\text{th}}$ unit cell are denoted by $u_{n,j}^1$ and $u_{n,j}^3$, respectively. The indices n and j denote the unit cell location in the x and y coordinates. Considering the x-directional displacement of m_1 to analyze the S0 wave mode propagating along the x direction, we use the following equations of motion:

$$m_1 \frac{\partial^2 u_{n,j}^1}{\partial t^2} = \alpha(u_{n+1,j}^1 + u_{n-1,j}^1 - 2u_{n,j}^1) + \delta(u_{n,j+1}^3 + u_{n,j}^3 - 2u_{n,j}^1) , \tag{S5a}$$

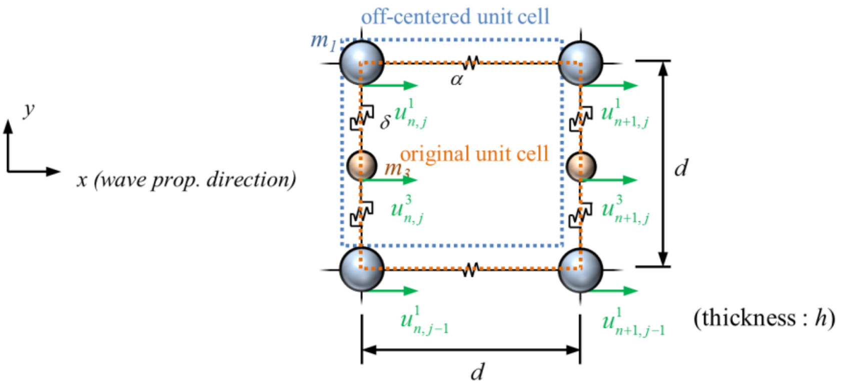

Fig. S2. The discrete mass-spring system corresponding to the metamaterial made of $\boldsymbol{C_m}$.

$$m_3 \frac{\partial^2 u^3_{n,j}}{\partial t^2} = \delta(u^1_{n,j} + u^1_{n,j-1} - 2u^3_{n,j}) . \tag{S5b}$$

Note that there is no need to consider displacements in the *y* axis because the *y*-displacements do not interact with the S0 wave mode (dominated by *x*-displacements of m_1). Also, the displacements $u^1_{n,j}$ and $u^3_{n,j}$ can be assumed not to vary along the *y* axis. Therefore, one can set $u^3_{n,j+1} = u^3_{n,j}$ and $u^1_{n,j+1} = u^1_{n,j}$.

Assuming time-harmonic wave motion at an angular frequency of ω, equation (S5) can be re-written as

$$-\omega^2 m_1 u^1_{n,j} = \alpha(\exp(ikd) + \exp(-ikd) - 2)u^1_{n,j} + 2\delta(u^3_{n,j} - u^1_{n,j}) , \tag{S6a}$$

$$-\omega^2 m_3 u^3_{n,j} = 2\delta(u^1_{n,j} - u^3_{n,j}) . \tag{S6b}$$

From equation (S6b),

$$u^3_{n,j} = \frac{2\delta}{2\delta - \omega^2 m_3} u^1_{n,j} . \tag{S7}$$

Substituting equation (S7) into equation (S6a) yields

$$-\omega^2 m_1 u^1_{n,j} = \alpha(\exp(ikd) + \exp(-ikd) - 2)u^1_{n,j} + \frac{2\delta m_3 \omega^2}{2\delta - \omega^2 m_3} u^1_{n,j} . \tag{S8}$$

From equation (S8), the following dispersion relation can be obtained for the system of $\boldsymbol{C_m}$:

$$-\omega^2(m_1 + \frac{2\delta m_3}{2\delta - \omega^2 m_3}) = \alpha(\exp(ikd) + \exp(-ikd) - 2)\,. \tag{S9}$$

To find an expression for the characteristic impedance, we write the force exerted on the $(n+1)^{\mathrm{th}}$ mass by the nth mass as

$$F = \alpha(u_{n,j}^1 - u_{n+1,j}^1) = \alpha(\exp(ikd) - 1)u_{n+1,j}^1\,. \tag{S10}$$

Using equation (S10), one can identify the characteristic impedance as

$$Z = -\frac{Fi}{\omega dh u_{n+1,j}^1} = \alpha\frac{(1 - \exp(ikd))i}{\omega dh}\,. \tag{S11}$$

Analytic investigation of the metamaterial with the unit cell $\boldsymbol{C}_k$

The equivalent mass spring system of the metamaterial with the unit cell $\boldsymbol{C}_k$ is plotted in Fig. S3. Writing up all equations of motion for m_1 and m_2,

$$m_1\frac{\partial^2 u_{n,j}^1}{\partial t^2} = -4\alpha u_{n,j}^1 + \alpha u_{n,j}^2 + \alpha u_{n-1,j}^2 + \alpha u_{n,j}^{2'} + \alpha u_{n-1,j}^{2'} + \gamma v_{n,j}^2 - \gamma v_{n-1,j}^2 - \gamma v_{n,j}^{2'} + \gamma v_{n-1,j}^{2'}\,, \tag{S12a}$$

$$m_2\frac{\partial^2 u_{n,j}^2}{\partial t^2} = -2\alpha u_{n,j}^2 + \alpha u_{n+1,j}^1 + \alpha u_{n,j}^1\,, \tag{S12b}$$

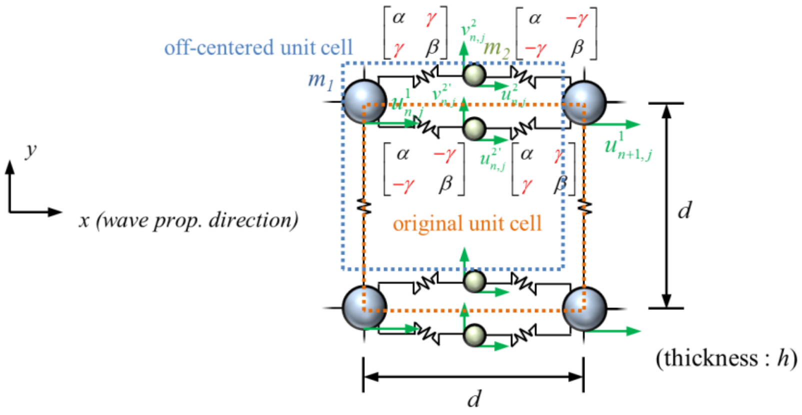

Fig. S3. The discrete mass-spring system corresponding to the metamaterial made of $\boldsymbol{C}_k$.

$$m_2 \frac{\partial^2 u_{n,j}^{2'}}{\partial t^2} = -2\alpha u_{n,j}^{2'} + \alpha u_{n+1,j}^1 + \alpha u_{n,j}^1 , \tag{S12c}$$

$$m_2 \frac{\partial^2 v_{n,j}^{2}}{\partial t^2} = -\gamma u_{n+1,j}^1 + \gamma u_{n,j}^1 - 2\beta v_{n,j}^2 , \tag{S12d}$$

$$m_2 \frac{\partial^2 v_{n,j}^{2'}}{\partial t^2} = \gamma u_{n+1,j}^1 - \gamma u_{n,j}^1 - 2\beta v_{n,j}^{2'} . \tag{S12e}$$

In equation (S12), the quantities with superscripts 2 and $2'$ are related to the upper and the lower mass m_2, respectively. Assuming time-harmonic wave motion, equations (S12) can be re-written as

$$\begin{aligned} -\omega^2 m_1 u_{n,j}^1 &= -4\alpha u_{n,j}^1 + \alpha(1+\exp(ikd))u_{n,j}^2 + \alpha(1+\exp(ikd))u_{n,j}^{2'} \\ &\quad + \gamma(1-\exp(ikd))v_{n,j}^2 - \gamma(1-\exp(ikd))v_{n,j}^{2'} \end{aligned} , \tag{S13a}$$

$$-\omega^2 m_2 u_{n,j}^2 = \alpha(1+\exp(-ikd))u_{n,j}^1 - 2\alpha u_{n,j}^2 , \tag{S13b}$$

$$-\omega^2 m_2 u_{n,j}^{2'} = \alpha(1+\exp(-ikd))u_{n,j}^1 - 2\alpha u_{n,j}^{2'} , \tag{S13c}$$

$$-\omega^2 m_2 v_{n,j}^2 = \gamma(1-\exp(-ikd))u_{n,j}^1 - 2\beta v_{n,j}^2 , \tag{S13d}$$

$$-\omega^2 m_2 v_{n,j}^{2'} = -\gamma(1-\exp(-ikd))u_{n,j}^1 - 2\beta v_{n,j}^{2'} . \tag{S13e}$$

Re-writing equations (S13 b-e) with respect to the displacement $u_{n,j}^1$ yields

$$u_{n,j}^2 = u_{n,j}^{2'} = \frac{\alpha(1+\exp(-ikd))}{2\alpha - \omega^2 m_2} u_{n,j}^1 = \frac{\alpha(\exp(ikd)+1)}{2\alpha - \omega^2 m_2} u_{n+1,j}^1 , \tag{S14a}$$

$$v_{n,j}^2 = -v_{n,j}^{2'} = \frac{\gamma(1-\exp(-ikd))}{2\beta - \omega^2 m_2} u_{n,j}^1 = \frac{\gamma(\exp(ikd)-1)}{2\beta - \omega^2 m_2} u_{n+1,j}^1 , \tag{S14b}$$

By substituting equations (S14a, b) to equation (S13a), one can finally obtain the dispersion equation as

$$-\omega^2 (m_1 + \frac{4\alpha m_2}{2\alpha - \omega^2 m_2}) = \left[\frac{2\alpha^2}{2\alpha - \omega^2 m_2} - \frac{2\gamma^2}{2\beta - \omega^2 m_2} \right] (\exp(-ikd) + \exp(ikd) - 2) . \tag{S15}$$

If the operating frequency of interest is assumed to be much lower than $2\alpha / m_2$ (this

situation is very typical because α is usually one order larger than β as in Table S1), one can assume that

$$2\alpha - \omega^2 m_2 \approx 2\alpha \tag{S16}$$

and equation (S16) can be simplified to

$$-\omega^2 (m_1 + 2m_2) = \left[\alpha - \frac{2\gamma^2}{2\beta - \omega^2 m_2} \right] (\exp(-ikd) + \exp(ikd) - 2) . \tag{S17}$$

To find the characteristic impedance for the system, the force exerted on the $(n+1)^{\text{th}}$ mass by the nth mass is obtained as

$$F = -(2\alpha u_{n+1,j}^1 - \alpha u_{n,j}^2 - \alpha u_{n,j}^{2'} + \gamma v_{n,j}^2 - \gamma v_{n,j}^{2'}) . \tag{S18}$$

Substituting equations (S14a, b) into equation (S18) yields

$$F = \left[-2\alpha + \frac{2\alpha^2 (1 + \exp(ikd))}{2\alpha - \omega^2 m_2} - \frac{2\gamma^2 (\exp(ikd) - 1)}{2\beta - \omega^2 m_2} \right] u_{n+1,j}^1 . \tag{S19}$$

Using the assumption in equation (S16), equation (S19) is simplified to

$$\begin{aligned} F &= \left[-2\alpha + \alpha(1 + \exp(ikd)) - \frac{2\gamma^2 (\exp(ikd) - 1)}{2\beta - \omega^2 m_2} \right] u_{n+1,j}^1 \\ &= \left[\alpha - \frac{2\gamma^2}{2\beta - \omega^2 m_2} \right] (\exp(ikd) - 1) u_{n+1,j}^1 \end{aligned} . \tag{S20}$$

Accordingly, the characteristic impedance becomes

$$Z = -\frac{Fi}{\omega d h u_{n+1,j}^1} = \left[\alpha - \frac{2\gamma^2}{2\beta - \omega^2 m_2} \right] \frac{(1 - \exp(ikd)) i}{\omega d h} . \tag{S21}$$

Numerical validations of the effective mass and stiffness

The validation of the effective mass and stiffness derived in the main manuscript can be made directly or indirectly. As an indirect way, one can compute the dispersion curve by using the one-dimensional dispersion equation (1) with the effective mass and stiffness in

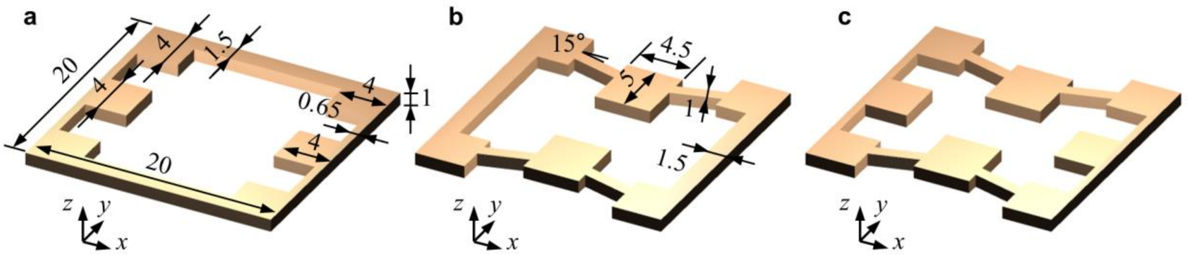

Fig. S4. Sketches of the elastic solid unit cells of (a) $\boldsymbol{C_m}$ (b) $\boldsymbol{C_k}$ and (c) $\boldsymbol{C_{mk}}$ with the specific dimensions. The numbers in the figure are all in the millimeter scale.

equations (4,8,10) and compare the curve with that obtained for the original solid unit cell shown in Fig. 1. The specific dimensions and geometries are given in Fig. S4. To extract the lumped parameters in equations (4,8,10), the mass and spring coefficients of the continuum structures in Fig. S4, one may attempt to derive formula to relate the geometric parameters to the mass and spring coefficients as done in Refs.[S1-S3]. Because of the complexity in geometry, we used the finite element method for accurate estimation. Table S1 lists the calculated mass and stiffness values for the three types of unit cells, which were calculated with 3-

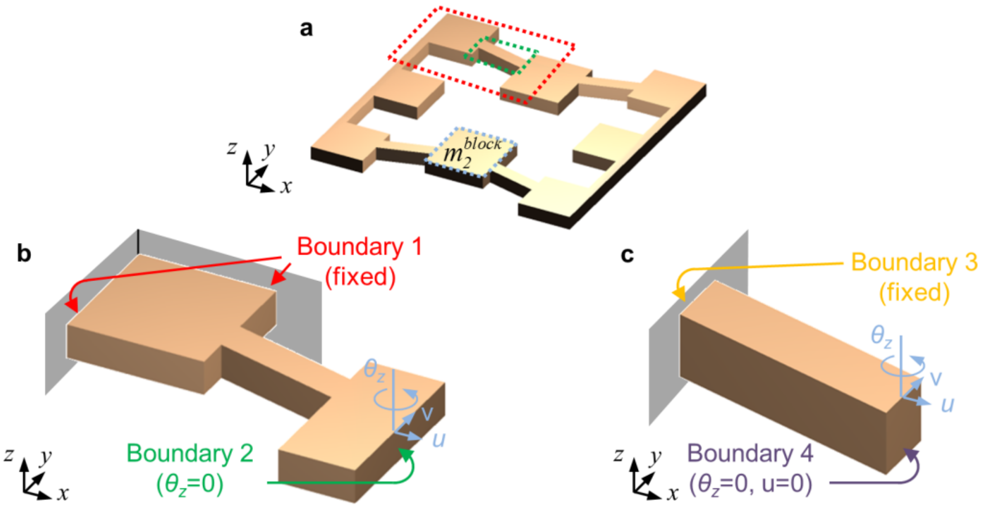

Fig. S5. (a) The unit cell of $\boldsymbol{C_{mk}}$, the finite element model to calculate (b) the spring coefficients α, β and γ, (c) the additional mass for the mass coefficient m_2, for the unit cell of $\boldsymbol{C_{mk}}$.

dimensional finite element simulations. For instance, the spring coefficients α, β and γ, and the mass coefficient m_2, for the unit cell of $\boldsymbol{C}_{\boldsymbol{mk}}$, are calculated with the finite element model shown in Fig. S5. We will briefly explain how these coefficients are calculated.

To calculate the spring coefficients α, β and γ, only the part marked as the red box in Fig. S5 (a) needs to be considered. Here, the fixed boundary conditions are imposed on Boundary 1 and $\theta_z = 0$ (no rotation about the *z* axis) is imposed on Boundary 2. These conditions are illustrated in Fig. S5 (b). The part in Fig. S5 (b) is discretized by the finite elements and we calculated *u* and *v*, the *x*- and *y*- directional displacements, at the center point of Boundary 2 by applying F_x and F_y, the *x*- and *y*- directional forces, independently. The calculated displacements are used to construct the compliance matrix $\mathbf{B}$ that relates the forces and displacements as $\mathbf{B}\begin{bmatrix} F_x \\ F_x \end{bmatrix} = \begin{bmatrix} u \\ v \end{bmatrix}$. Finally, the spring coefficients α, β and γ can be evaluated by taking the inverse of $\mathbf{B}$, resulting in

$$\begin{bmatrix} \alpha & -\gamma \\ -\gamma & \beta \end{bmatrix} = \mathbf{B}^{-1}. \tag{S22}$$

	**Metamaterial with *x*-resonating part**	**Metamaterial with *y*-resonating part**	**Metamaterials with *x*- and *y*-resonating parts**
α	1.24e4 kN/m	1.02e4 kN/m	1.02e4 kN/m
β	1.15e3 kN/m	1.23e3 kN/m	1.23e3 kN/m
γ	1.10e-3 kN/m	2.44e3 kN/m	2.43e3 kN/m
δ	1.04e3 kN/m	3.26e3 kN/m	1.04e3 kN/m
m_1	2.52e-4 kg	2.53e-4 kg	2.21e-4 kg
m_2	1.85e-5 kg	6.71e-5 kg	6.71e-5 kg
m_3	9.59e-5 kg	4.70e-5 kg	9.59e-5 kg

Table S1. The estimated values of mass and stiffness elements of the discrete mass-spring models corresponding to the unit cells shown in Fig. S4. The calculations were performed by the 3D finite element analysis for accurate evaluation.

To calculate the mass coefficient m_2, one can simply choose $m_2 = m_2^{block}$ where m_2^{block} denotes the mass of the block marked as the blue box in Fig. S5 (a). For more precise estimation, the mass of the slender beam part, marked as the green box in Fig. S5 (a), can be additionally considered because the beam part serves not only as stiffness but also as mass[S3]. The beam part in consideration is illustrated as Fig. S5 (c). To accurately evaluate the additional mass coefficient from the slender beam structure, which will be denoted as m_2^{beam}, the most accurate method is to perform the free vibration analysis, yielding the lowest bending eigenfrequency of $\omega_{bending}^{beam}$. Because the bending stiffness of the beam segment β^{beam} can be evaluated by applying a force in the y direction at the center point of Boundary 4, one can evaluate m_2^{beam} from the well-known eigenfrequency of a single degree-of-freedom vibration as $m_2^{beam} = \beta^{beam} / (\omega_{bending}^{beam})^2$. Finally, we find $m_2 = m_2^{block} + 2m_2^{beam}$. Note that here, m_2^{beam} is smaller than one tenth of m_2^{block} and m_2^{beam} can be actually ignored.

Fig. S6 compares the dispersion curves where the dispersion curves for the original solid unit cells were computed by the 3-dimensional finite element method[S4]. Excellent agreements

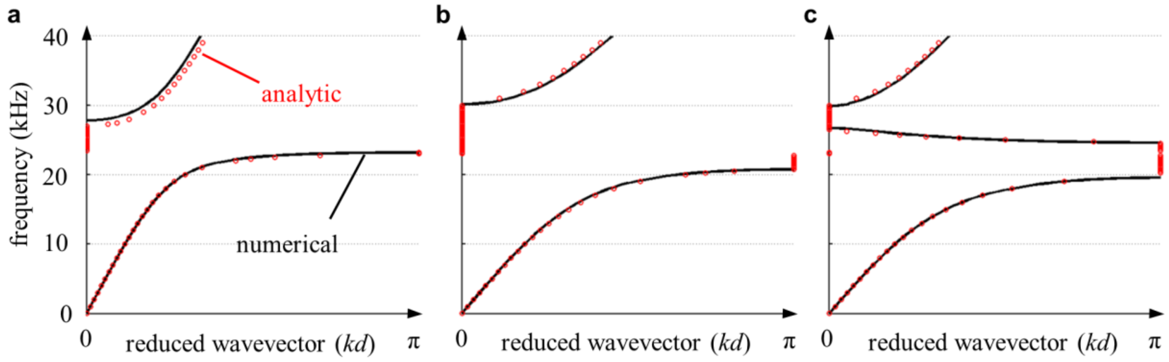

Fig. S6. Comparison of the dispersion curves numerically calculated from the original elastic solid metamaterials (in black solid lines) and from the effective mass and stiffness values (in red circles) calculated by equation (1), equivalently, equations (4, 8, 10). The results are for the metamaterials made of (a) $\boldsymbol{C}_{\boldsymbol{m}}$ (b) $\boldsymbol{C}_{\boldsymbol{k}}$ and (c) $\boldsymbol{C}_{\boldsymbol{mk}}$.

between the two results are observed for all three cases corresponding to $\boldsymbol{C}_m$, $\boldsymbol{C}_k$ and $\boldsymbol{C}_{mk}$, validating the derived effective mass and stiffness. This clearly shows that the S0 wave mode which is a 3-dimensional wave phenomenon can be accurately described with the proposed two-dimensional mass-spring system.

A more direct way to validate the derived effective mass and stiffness is to estimate the effective parameters by the retrieval method developed for elastic metamaterials[S5]. The details of the retrieval method will not be given here, but the complex reflection and transmission coefficients of a metamaterial layer consisting of one of the metamaterials in Fig. S4 were numerically calculated with 3-dimensional finite element simulations. Then, the effective density and stiffness can be retrieved with the retrieval method. Fig. S7 compares the retrieved values and those obtained by equations (4, 8, 10). For all cases, good agreements were found, validating our theory.

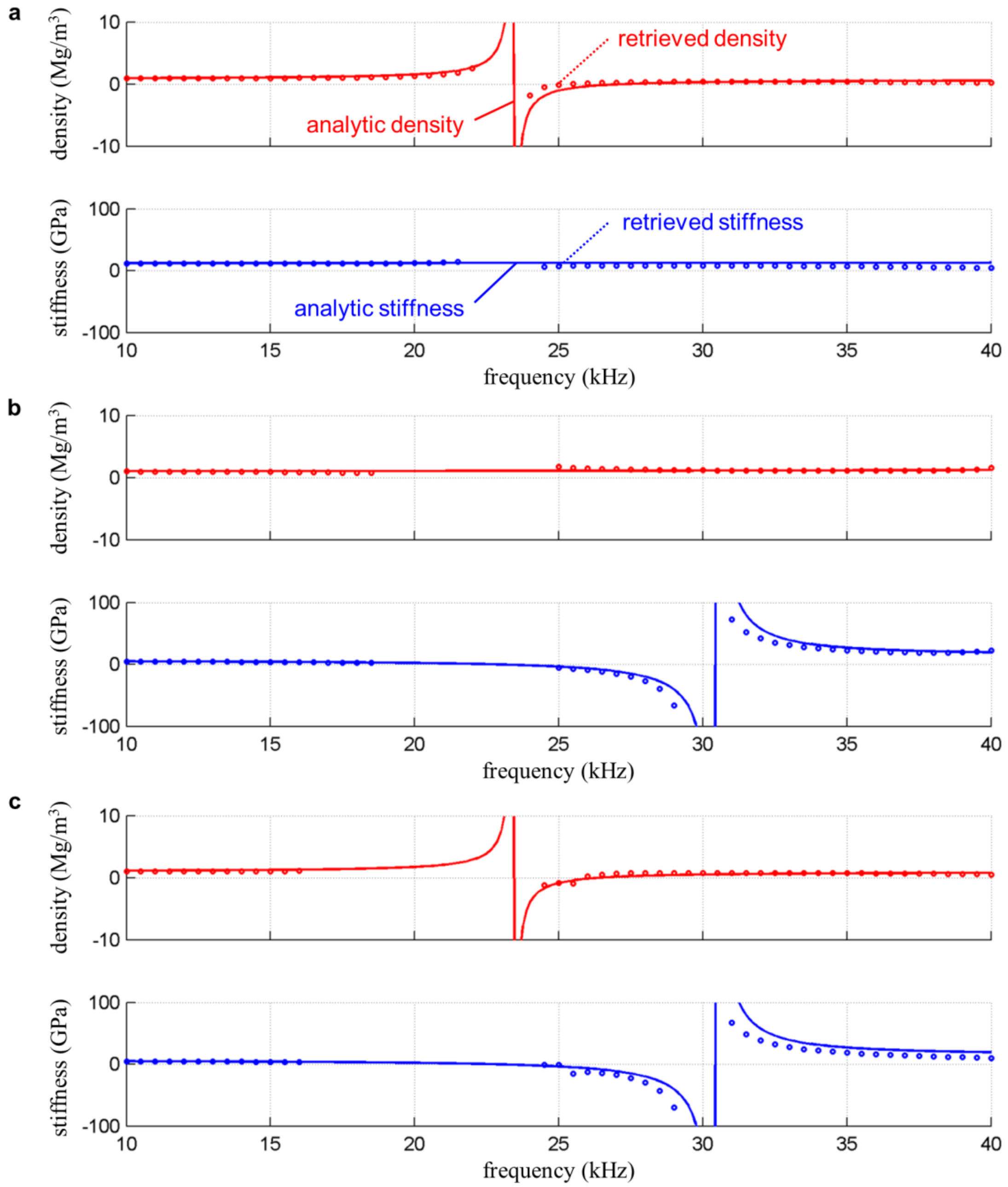

Fig. S7. Comparison of the effective mass and stiffness values numerically calculated by the retrieval method applied to the original continuum metamaterials (in circles) and those by equations (4, 8, 10) (in solid lines). The results are for the metamaterials made of (a) $\boldsymbol{C}_m$ (b) $\boldsymbol{C}_k$ and (c) $\boldsymbol{C}_{mk}$, respectively.

Numerical simulation to investigate phase velocities in the metamaterials

Fig. S8 (a) shows the model used to numerically investigate phase velocities inside the metamaterial layer. The metamaterial layer consisting of 4 $\boldsymbol{C}_{mk}$ unit cells is inserted between two aluminum plates. The plates and layer are discretized by finite elements for numerical analysis. For the simulations, the S0 sinusoidal waves of 15, 25 and 35 kHz are incident from the left aluminum plate and the *x*-directional displacements u_j (j =*A*, *B* and *C*) are measured where *A*, *B* and *C* denote points inside the metamaterial layer. The theory predicts that the metamaterials made of $\boldsymbol{C}_{mk}$ should exhibit positive phase velocities at 15 and 35 kHz, but a negative phase velocity at 25 kHz.

Figs. S8 (b-d) show the simulation results for the displacements u_A, u_B and u_C at 15, 25 and 35 kHz, respectively. At 15 and 35 kHz, the crests move forward along the positive *x* direction as the wave propagates. At 25 kHz, on the other hand, the crests move backwards

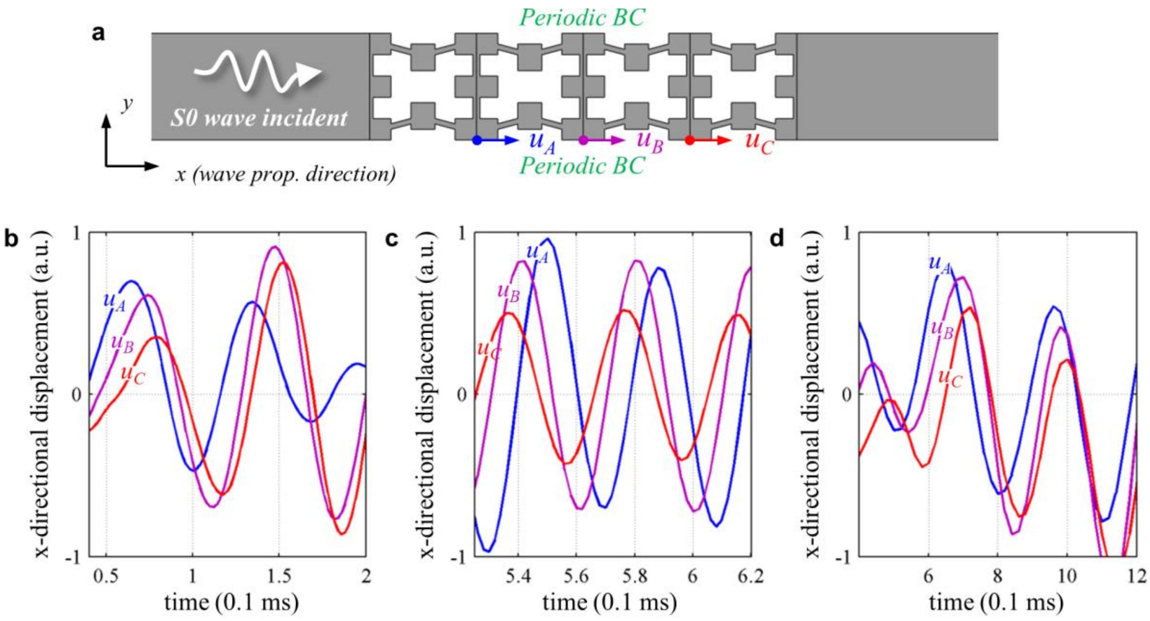

Fig. S8. (a) Numerical simulation modeling for the metamaterials made of $\boldsymbol{C}_{mk}$, and the measured displacement values at the frequency of (b) 15, (c) 25 and (d) 35 kHz.

along the negative x direction as the wave propagates along the positive x direction. This means that the phase velocity at 25 kHz is negative. The phase velocities measured from the numerical simulations are found to be 1739, -2500 and 3333 m/s while the phase velocities calculated from the dispersion curve are 1729, -2353 and 3294 m/s for the frequency of 15, 25 and 35 kHz, respectively. The numerical simulations confirm the validity of the analytic prediction, including the formation of the negative phase velocity at 25 kHz.

Experimental setting for the metamaterials

Fig. S9 illustrates the schematic figure of the experimental setup. The imbedded metamaterials in the base aluminum plate are fabricated by the waterjet cutting process. The dimensions of the base plate are 2 m in width, 1.2 m in height and 1 mm in thickness. The imbedded metamaterial system fabricated in the middle of the base plate consists of 4×25 unit cells with 4 unit cells along the propagating x direction. Fig. S10 shows the photos of the fabricated metamaterials made of $\boldsymbol{C_m}$, $\boldsymbol{C_k}$ and $\boldsymbol{C_{mk}}$. The traction-free top and bottom surfaces ensure the formation of guided waves along the x direction[S6]. The actuator and sensor locations for wave experiments are shown in Fig. S9. Patch-type piezoelectric transducers (thickness: 1 mm, radius: 1.2 cm) were installed 5 cm away from the boundaries of the metamaterial system to generate and measure the S0 wave mode.

The detailed experimental procedure is as follows. First, the actuation signal was generated by a function generator (33250A, Agilent Technologies Inc., Santa Clara, CA). The modulated Gaussian pulse, or the Gabor pulse, was used as the actuation signal which was generated by

$$\exp(-\frac{f^2}{2G_s^2}t^2)\cos(2\pi ft)\,. \tag{S23}$$

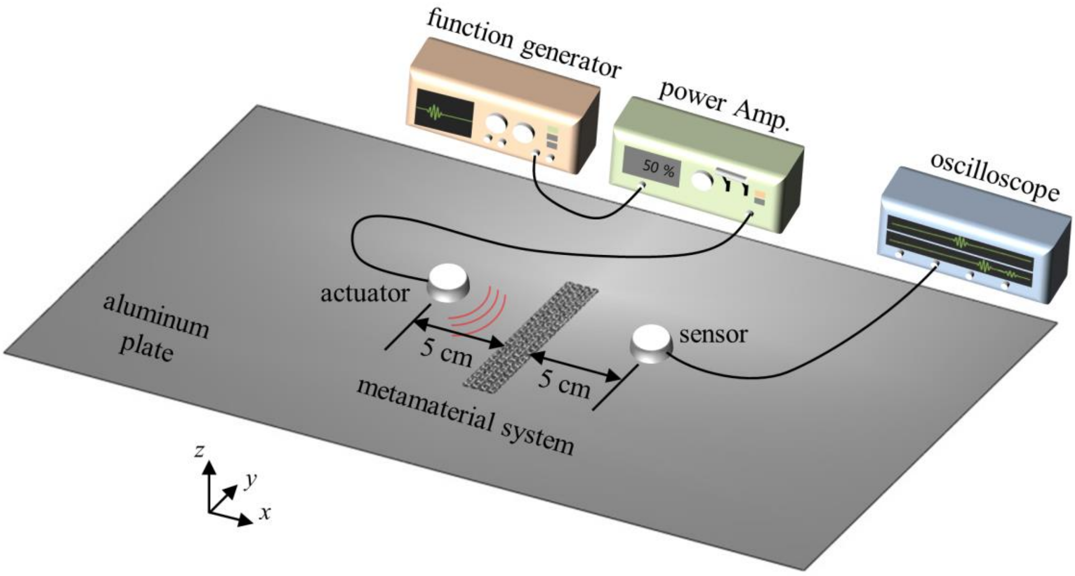

Fig. S9. Schematic figure of the experimental setup.

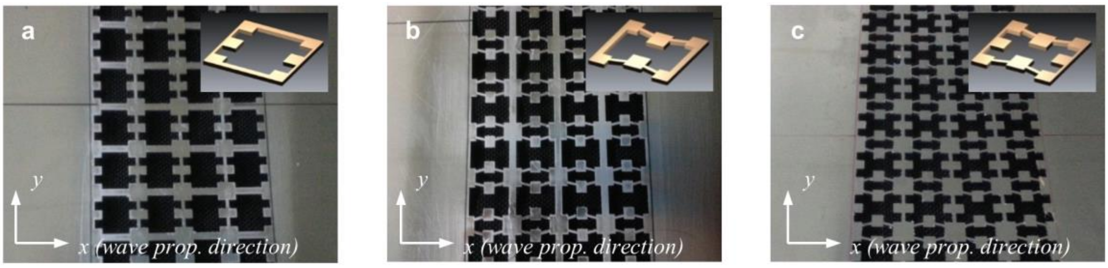

Fig. S10. Photos of the fabricated metamaterials made of (a) $\boldsymbol{C_m}$ (b) $\boldsymbol{C_k}$ and (c) $\boldsymbol{C_{mk}}$, respectively. The experimental results in Fig. 6 are based on the fabricated metamaterials illustrated here.

In equation (S23), G_s is a factor that controls the time spread of the pulse and f is center frequency of the pulse. In our experiments, G_s was set to be 2.75 and the center frequencies (f) were chosen to be 15, 25 and 35 kHz. The actuated signal was amplified by a power amplifier (AG1017L, T&C Power Conversion, Rochester, NY) and sent to the actuating piezoelectric transducer. The wave transmitted through the metamaterial layer system was measured by the receiving piezoelectric transducer and the signal from the receiving transducer was recorded by an oscilloscope (WaveRunner 104MXi-A, LeCroy, Chestnut

Ridge, NY). The excitation and measured signals at 35 kHz are shown in Figs. S11 (a,b). Measured signals were post-processed before they were compared with simulation results.

For the comparison, we used the arrival time t_a defined as

$$t_a = \frac{d^{al}}{v_g^{al}\big|_{S0}} + \frac{4d_{unit}^{meta}}{v_g^{meta}\big|_{S0}} = \frac{0.1}{v_g^{al}\big|_{S0}} + \frac{0.08}{v_g^{meta}\big|_{S0}} \tag{S24}$$

where the superscripts '*al*' and '*meta*' stand for aluminum and metamaterial. The symbol d_{al} denotes the sum of the distances from the actuating and receiving transducers to the metamaterial boundaries. The symbol d_{unit}^{meta} represents the size of the metamaterial unit cell

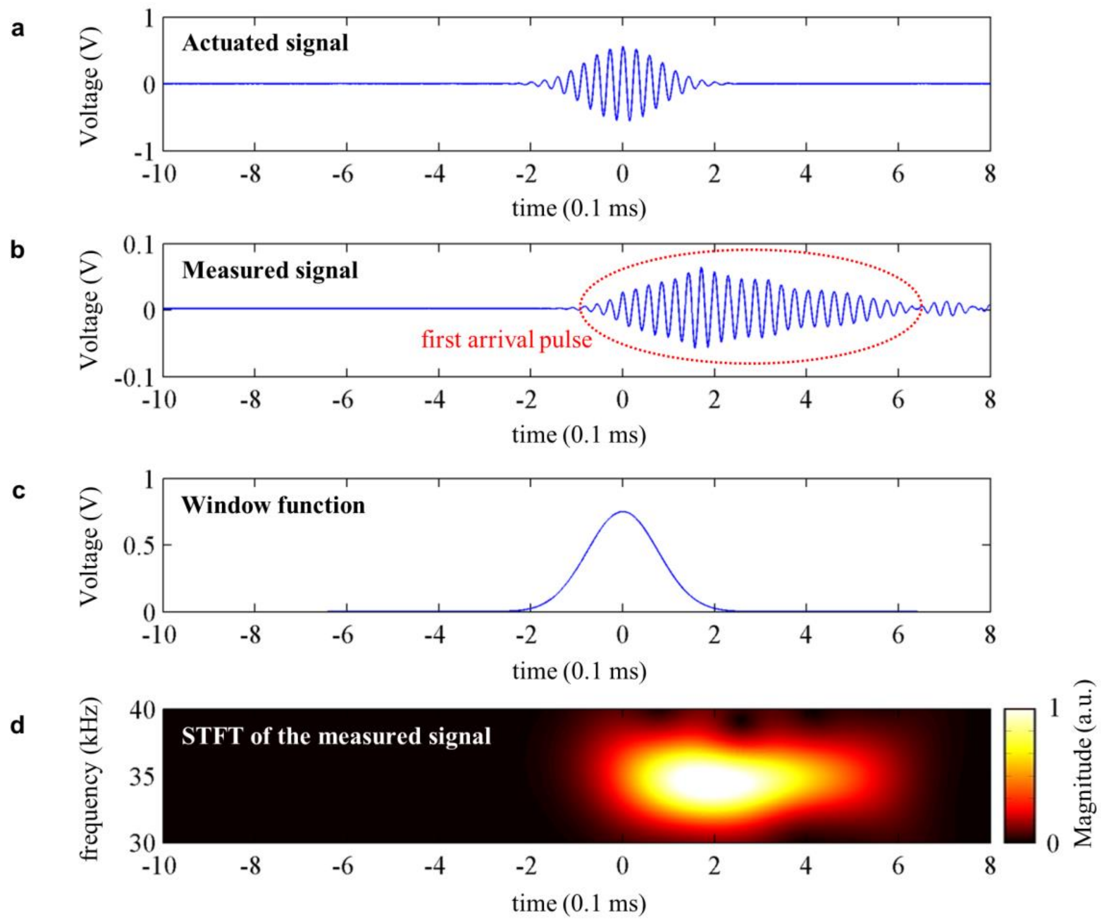

Fig. S11. Experimental signals obtained for the excitation frequency of $f = 35$ kHz. (a) Actuated signal, (b) measured signal, (c) the Gaussian window function used for the STFT and (c) the STFT of the first arrival pulse in (b).

in the x direction. The experimental arrival time t_a can be estimated from the experimental data by performing the short-time Fourier transformation (STFT) (see, e.g., Mallat[S7]) of the first arrival pulse (as marked in Fig. S11 (b)). The short-time Fourier transform $SV(\tau,\omega)$ of $V(t)$ is defined as

$$SV(\tau,\omega)=\int_{-\infty}^{\infty}V(t)g(t-\tau)\exp(-i\omega t)dt \tag{S25}$$

where $g(t)$ is a real symmetric window function, τ denotes the amount of the translation of $g(t)$ in time and ω, the angular modulation frequency. The selected window function $g(t)$ is the Gaussian window plotted in Fig. S11 (c). Fig. S11 (d) plots the absolute value $|SV(\tau,\omega)|$ for varying τ (representing the horizontal axis) and ω (representing the vertical axis). Note that $|SV(\tau,\omega)|^2$ is usually called the spectrogram denoting the energy of $V(t)$ in the time-frequency neighbor of (τ,ω). The level of color at (τ,ω) in Fig. S11 (d) corresponds to the magnitude of the output voltage in the neighbor of (τ,ω). If ridges are identified from $|SV(\tau,\omega)|$ or $|SV(\tau,\omega)|^2$, the information of the arrival time of a specific harmonic component can be extracted. (The analysis of dispersive waves by the STFT may be found in Ref.[S8]) For instance, t_a of the S0 wave can be found by reading the local maxima in the STFT plot in Fig. S11 (d). On the other hand, one can numerically calculate the arrival time t_a by using the group velocity of the S0 wave mode $v_g^{meta}\big|_{S0}$ in the metamaterial and that in the base aluminum plate, $v_g^{al}\big|_{S0}$.

Verification of independent tunability: Experiments and findings

To verify the independent tunability of the proposed elastic metamaterial, additional experiments were performed. Starting from the original $\boldsymbol{C}_{mk}$ configuration, the x- and y-

Unit cell type	C_{mk} (original)	$C_{\tilde{m}k}$ (change of the x-resonating part)	$C_{m\tilde{k}}$ (change of the y-resonating part)
Configuration	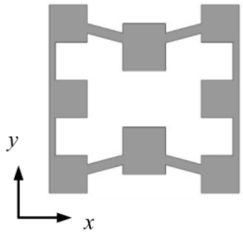	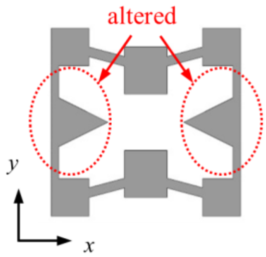	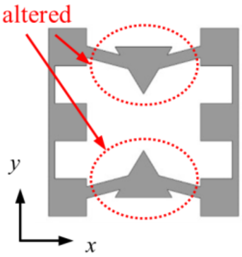
f_x Resonance freq. in x	23.13 kHz	32.19 kHz	23.13 kHz
f_y Resonance freq. in y	30.08 kHz	30.08 kHz	38.59 kHz
Negative density range	23.05 ~ 28.33 kHz	almost identical<br>32.30 ~ 37.25 kHz	22.60 ~ 27.34 kHz
Negative stiffness range	20.96 ~ 30.23 kHz	almost identical<br>20.38 ~ 30.14 kHz	30.87 ~ 38.66 kHz

Table S2. Comparison of C_{mk} and $C_{\tilde{m}k}$ against $C_{m\tilde{k}}$.

resonating parts are separately varied to make $C_{\tilde{m}k}$ and $C_{m\tilde{k}}$ unit cells. They are compared against C_{mk} in Table S2. The objective to perform experiments with $C_{\tilde{m}k}$ and $C_{m\tilde{k}}$ is to show that the negative density or the negative stiffness can be altered alone without affecting its counterpart, the negative stiffness or the negative density, respectively.

First, a new metamaterial made of $C_{\tilde{m}k}$ shown in Fig. S12 (a) is considered. In $C_{\tilde{m}k}$, the x-resonating part is replaced by a new resonator the resonance frequency of which is moved to 30.08 kHz while the y-resonating part is the same as that of C_{mk}. Therefore, two resonance gaps around 25 kHz (due to the negative stiffness) and 35 kHz (due to the negative density) are formed in the metamaterial made of $C_{\tilde{m}k}$. The experimental results in Fig. S12

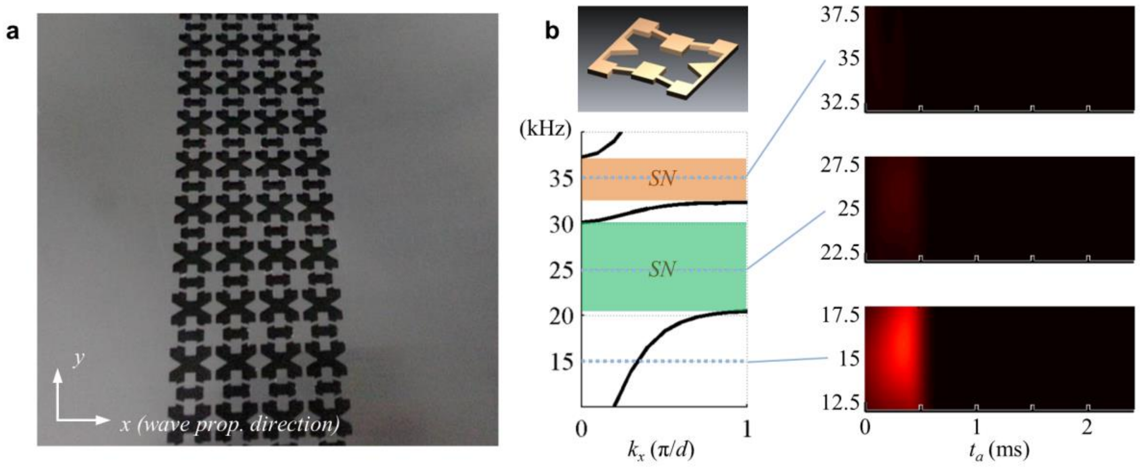

Fig. S12. (a) The picture of the metamaterial made of $C_{\tilde{m}k}$ for which only the *x*-resonance frequency is changed to around 32.19 kHz, (b) the dispersion curve and experimental results of the metamaterial made of $C_{\tilde{m}k}$.

(b) show that there is no S0 wave mode transmission around 25 and 35 kHz, indicating that the negative density region is moved to around 35 kHz. On the other hand, the negative stiffness region around 25 kHz is virtually unaltered; the range of the resonance gap due to the negative stiffness for the metamaterial made of $C_{\tilde{m}k}$ is 20.38 ~ 30.14 kHz while that for the metamaterial made of C_{mk} is 20.96 ~ 30.23 kHz. This result confirms independent tuning of the negative density without affecting the negative stiffness of C_{mk}.

Now, let us consider a new metamaterial made of $C_{m\tilde{k}}$ shown in Fig. S13 (a). In this case, the resonance gap due to the negative stiffness is formed around 35 kHz while the resonance gap due to the negative density remains unaltered in comparison with the metamaterial made of C_{mk}; see Fig. S13 (b). The experimental results confirm that the range of the resonance gap due to the negative density for the metamaterial made of $C_{m\tilde{k}}$ is 22.60 ~ 27.34 kHz while that for the metamaterial made of C_{mk} is 23.05 ~ 28.33 kHz. This result confirms independent tuning of the negative stiffness without altering the negative density of C_{mk}. From the experimental results in Figs. S12 and S13, negative density and stiffness are found

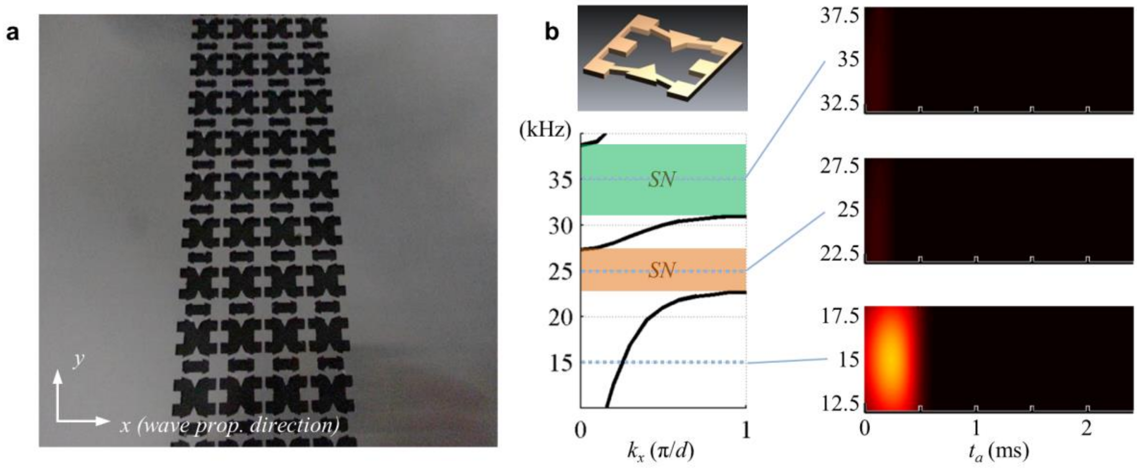

Fig. S13. (a) The picture of the metamaterial made of $\boldsymbol{C}_{\boldsymbol{m}\tilde{\boldsymbol{k}}}$ for which only the *y*-resonance frequency is changed to 38.59 kHz, (b) the dispersion curve and experimental results of the metamaterial made of $\boldsymbol{C}_{\boldsymbol{m}\tilde{\boldsymbol{k}}}$.

to be independently tunable.

References

S1 Wang, Y. F., Wang, Y. S. & Su, X. X. Large bandgaps of two-dimensional phononic crystals with cross-like holes. *J. Appl. Phys.* **110**, 113520 (2011).

S2 Wang, Y. F. & Wang, Y. S. Complete bandgap in three-dimensional holey phononic crystals with resonators. *J. Vib. Acoust.* **135**, 041009 (2013).

S3 Oh, J. H., Hong, M. S. & Kim, Y. Y. A truly hyperbolic elastic metamaterial lens. *Appl. Phys. Lett.* **104**, 073503 (2014).

S4 Langlet, P., Hladky-Hennion, A.-C. & Decarpigny, J.-N. Analysis of the propagation of plane acoustic waves in passive periodic materials using the finite element method. *J. Acoust. Soc. Am.* **98**, 2792-2800 (1995).

S5 Lee, H. J. *Effective-property characterization of elastic metamaterials for advanced wave tailoring.* (Ph. D. dissertation, Seoul National University, 2014).

S6 Graff, K. F. *Wave motion in elastic solids* (Dover Publications, Inc., 1991).

S7 Mallat, S. *A wavelet tour of signal processing*. (Academic Press, 1999).

S8 Hong, J. C., Sun, K. H. & Kim, Y. Y. Dispersion-based short-time fourier transform applied to dispersive wave analysis. *J. Acoust. Soc. Am.* **117**, 2949 -2960 (2005).
